# Nutriments: Integrating Patient-Centered Nutrition Education for Medical Students within a Clinical Presentation Framework

**DOI:** 10.1007/s40670-026-02791-8

**Published:** 2026-06-17

**Authors:** Samantha Gaidula, Savanna Shaw, Sonia Lobo, Sireesha Mamillapalli, Gabi Waite

**Affiliations:** Department of Medical Education, Geisinger Commonwealth School of Medicine, Scranton, PA USA

## Abstract

“Nutriments” a standardized microlearning resource focused on integrating nutrition into clinical care, was developed to address identified gaps in medical nutrition education. Surveyed medical students at Geisinger Commonwealth School of Medicine reported improved understanding and support for adoption, indicating Nutriments’ potential to strengthen curricula and enhance national medical education.

**Supplementary Information:**

The online version contains supplementary material available at https://doi.org/10.1007/s40670-026-02791-8.

## Innovation

Evidence consistently shows that current medical education inadequately prepares physicians in nutrition disease management [1]. Across institutions, medical students overwhelmingly agree that nutrition education is essential, yet many report dissatisfaction with both the quantity and quality of instruction [1]. A study at Geisinger Commonwealth School of Medicine (GCSOM) revealed low self-perceived nutritional competency among most students, with insufficient formal education being cited as the primary barrier to effective counseling [2]. Nationally, these perceptions reflect persistent gaps in medical curricula, where nutrition content remains fragmented, inconsistently assessed, or insufficiently integrated into clinical training. As a result, graduates may recognize the importance of nutrition yet lack the confidence or skills to translate this knowledge into patient care.

To address these deficiencies and align with competency-based expectations [3], we aimed to enhance GCSOM’s Total Health Curriculum (THC) by developing a standardized, adaptable nutrition education tool. This resource, termed Nutriment, was designed to reinforce core pathophysiology while introducing patient-centered nutrition strategies that can be readily applied in clinical settings. Nutriments were intentionally structured to complement any nationally accredited medical curriculum and were provided as an optional resource within GCSOM’s student learning platform.

GCSOM’s THC is organized into three phases: Phase 1 consists of 18 months of didactic coursework that establishes biomedical and clinical foundations; Phase 2 includes 12 months of core clinical rotations; and Phase 3 emphasizes career differentiation through advanced clinical experiences such as sub-internships and electives. To align with this model of curriculum, we initially developed three Nutriments for the Phase 1 curricula focused on phenylketonuria, gout, and celiac disease, conditions selected for their relevance across all phases of training, frequent appearance in assessments, and clinical importance (Supplementary Material 1).

Nutriments were created using Canva, an open-access graphic design platform, and designed for five-minute comprehension to reduce cognitive load while preserving educational depth guided by the microlearning paradigm. Each Nutriment included a brief disease overview and clinical presentation, followed by management strategies, a suggested grocery list, a sample day of eating, and condition-specific nutritional considerations. All content was reviewed by a registered dietitian, ensuring accuracy and iterative refinement. Nutriments were piloted in Fall 2025 as an optional resource alongside required Phase 1 curricular content.

To assess student perceptions of Nutriments, we administered an anonymous, voluntary electronic survey to all four classes (M1–M4) during Fall 2025. The 14-item survey included closed- and open-ended questions evaluating usefulness, clarity, and applicability. Eighty-four students responded; however, because questions were optional, item-level responses ranged from 36 to 50, as not all respondents answered every question. Percentages reported in Table 1 were calculated per item response count.

**Table 1:** Student perception of Nutriments as a supplemental learning tool for nutrition education. Results from an anonymous electronic survey administered to all four classes (M1-M4) in Fall 2025 assessing perceived usefulness, clarity, and applicability of Nutriments. Data are presented as number (percentage) of respondents for each item, with percentages calculated based on the number of students responding to that specific question (item-level denominators vary due to optional responses). Not all survey questions are shown in the table, only selected closed-ended survey items are shown. Participant responses to open-ended survey questions were analyzed thematically

Survey Question (Number of Respondents)	No. (%)
Q1. Did the Nutriments that you reviewed for PKU, Gout and Celiac Disease enhance your understanding of the role of nutrition in disease management? (*N* = 49)	
Yes	37 (76%)
Not Sure	8 (16%)
No	4 (8%)
Q2. Were you already familiar with the nutrition information provided before reading these nutriments? (*N* = 50)	
Yes	8 (16%)
Some of it	37 (74%)
No	5 (10%)
Q4. Do you feel that any important information is missing from the Nutriments? (*N* = 42)	
Yes	4 (10%)
Not Sure	19 (45%)
No	19 (45%)
Q6. If Nutriments were included as optional resources within their associated disease-specific podcast modules in Phase 1, would you use them to enhance your nutrition knowledge? (*N* = 40)	
Yes	33 (83%)
Not Sure	5 (13%)
No	2 (5%)
Q10. Was the information presented in a logical and easy-to-follow format? (*N* = 37)	
Yes	34 (92%)
Not Sure	3 (8%)
No	0 (0%)
Q11. Were the language and depth of information appropriate for a medical student’s level? (*N* = 37)	
Yes	35 (95%)
Not Sure	2 (5%)
No	0 (0%)
Q13. Do you feel that these Nutriments would enhance GCSOM’s Total Health Curriculum? (*N* = 36)	
Yes	30 (83%)
Not Sure	5 (14%)
No	1 (3%)

Most students indicated support for Nutriments as a learning tool. 76% indicated that their understanding of nutrition’s role in disease management was improved. 83% stated they would use Nutriments to supplement required coursework, and 82% anticipated using them during clinical rotations or patient counseling. When asked which components were most beneficial, 60% selected the grocery list and 53% selected the pathophysiology summary. 92% agreed that the layout was logical and easy to follow, and 95% felt the level of detail was appropriate for medical trainees. Most respondents (83%) agreed that Nutriments would enhance GCSOM’s THC by connecting nutrition content to direct patient care.

Open-ended responses highlighted the value of translating nutrition content into realistic clinical guidance. Students described Nutriments as “practical,” “clinically relevant,” and useful for understanding “exactly what to tell a patient” regarding dietary changes. Several emphasized that nutrition-focused materials filled a recognized gap in undergraduate medical education.

These findings demonstrate student support for Nutriments as a standardized tool for integrating nutrition education into medical training, although generalizability is limited due to the low response rate (18%) across all four classes. Student perceptions suggest that Nutriments complement existing disease-focused content rather than compete with it, reinforcing foundational biomedical knowledge while translating learning into practical strategies. This aligns with national calls for improved integration of clinically relevant nutrition education within undergraduate medical curricula [3].

Several design features contributed to positive reception: a concise, visually engaging format minimized cognitive burden; emphasis on conditions commonly encountered ensured curricular alignment; and inclusion of patient-centered tools addressed counseling skills students consistently report as lacking [2]. The adaptability of Nutriments positions them as a scalable model for institutions seeking to strengthen nutrition education in alignment with evolving national expectations outlined by the Association of American Medical Colleges [3].

At GCSOM, we plan to expand Nutriments throughout the THC framework. Through early training intervention, Nutriments have the potential to enhance physician preparedness and ultimately improve patient care outcomes.

## Supplementary Information

Below is the link to the electronic supplementary material.

Supplementary Material 1
