# Supplementary material for: Nutriments: Integrating Patient-Centered Nutrition Education for Medical Students within a Clinical Presentation Framework
Source: Med Sci Educ. 2026 Jun 17;36(4):1923–5. doi: 10.1007/s40670-026-02791-8 (PMC13582725; doi:10.1007/s40670-026-02791-8)
Supplement: Supplementary file 1 [file 40670_2026_2791_MOESM1_ESM.pdf]

POM Block

A GUIDE FOR PHENYLKETONURIA

Authors: Samantha Gaidula and Savanna Shaw

What is Phenylketonuria?

Phenylketonuria (PKU) is an autosomal recessive disorder that causes the body to be unable to convert phenylalanine to tyrosine due to a deficiency in the enzyme phenylalanine hydroxylase (PAH) or a deficiency in BH4 (tetrahydrobiopterin). Phenylalanine then builds up in the blood, body tissues, and most importantly, the brain. This can lead to intellectual disability, seizures, behavioral issues, and skin related findings[1].

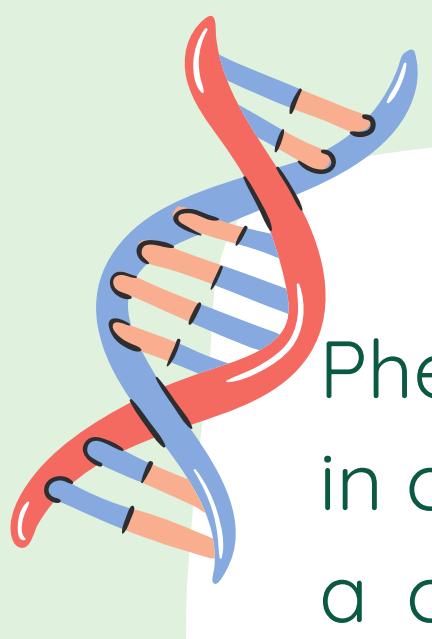

Disease Management

Phenylalanine should be avoided in patients with PKU, especially in childhood when the brain is rapidly developing . Phenylalanine is a common amino acid found in meat, dairy, seafood, nuts, soy beans, peas, beer, and artificial sweeteners. Patients with PKU may need to cut out many of these foods or eat them in limited quantities to avoid toxic buildup of phenylalanine. Blood levels of phenylalanine are often suggested to be kept at 120-360 μmol/L for children less than 12 and pregnant patients, while patients 12 and older should maintain a blood level of 120 to 600 μmol/L[2].

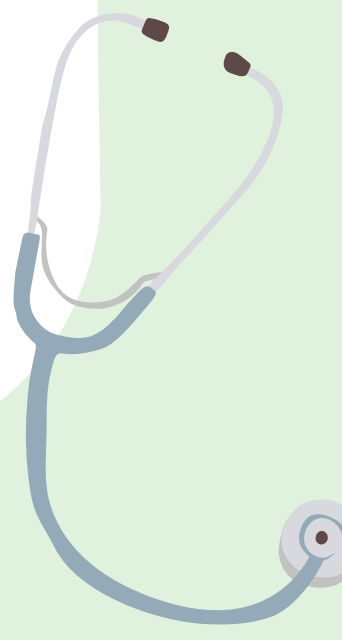

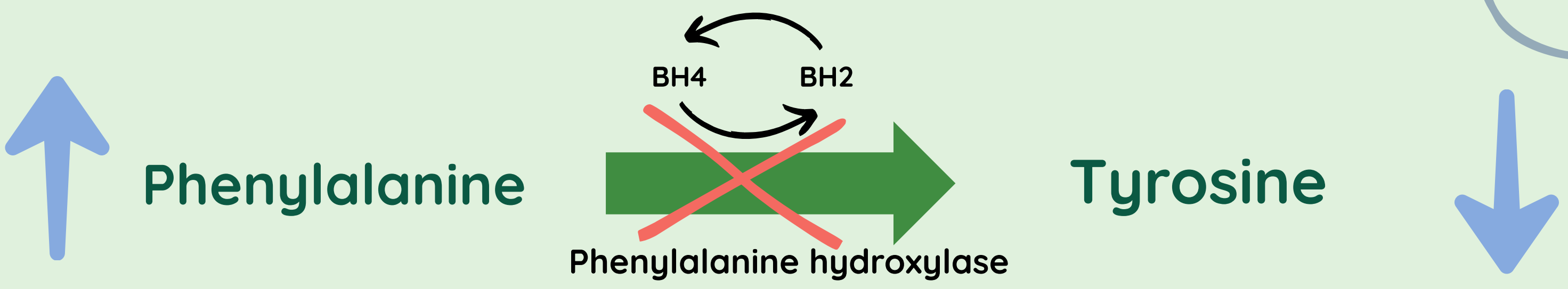

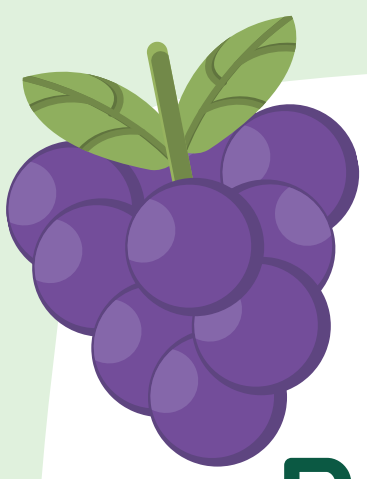

Grocery List Suggestions

*must check specific phenyalanine content of each

Recommended:	Limit:
fruits and vegetables	meat and poultry
butter and oils	lentils and beans
pastas and breads	aspartame (sweetener)

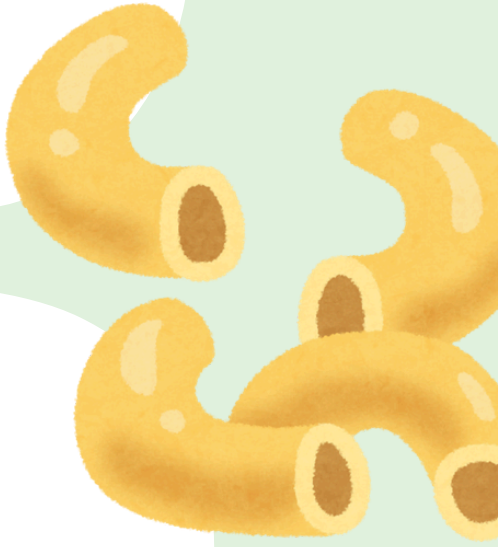

Recipe for Success

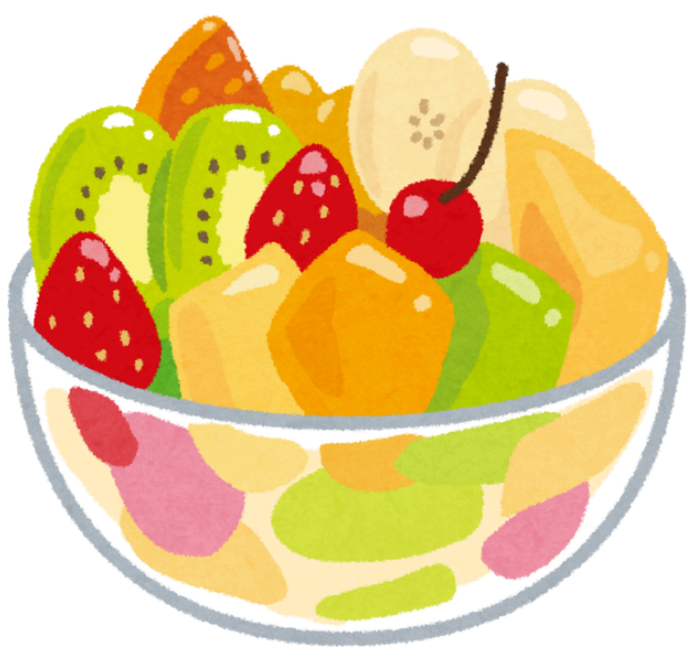

Breakfast: fruit smoothie with honey and coconut milk with a side of toast

Lunch: pasta salad with cucumber, peppers and cherry tomatoes with olive oil and lemon

Dinner: stir-fried vegetables with low-protein rice

A Word on Protein Substitutes

Protein substitutes are phenylalanine-free amino acid supplements that can be given as capsules, liquids, powders or bars. A protein substitute should be given at each meal to maintain energy/protein requirements as directed by your physician.

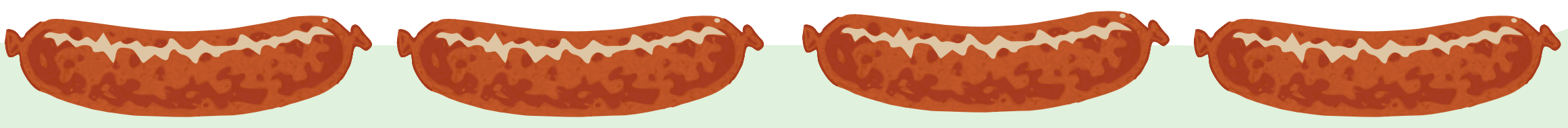

1. van Spronsen FJ, Blau N, Harding C, Burlina A, Longo N, Bosch AM. Phenylketonuria. Nat Rev Dis Primers. 2021 May 20;7(1):36. doi: 10.1038/s41572-021-00267-0. PMID: 34017006; PMCID: PMC8591558.
2. MacDonald A, van Wegberg AMJ, Ahring K, Beblo S, Bélanger-Quintana A, Burlina A, Campistol J, Coşkun T, Feillet F, Giżewska M, Huijbregts SC, Leuzzi V, Maillot F, Muntau AC, Rocha JC, Romani C, Trefz F, van Spronsen FJ. PKU dietary handbook to accompany PKU guidelines. Orphanet J Rare Dis. 2020 Jun 30;15(1):171. doi: 10.1186/s13023-020-01391-y. Erratum in: Orphanet J Rare Dis. 2020 Sep 1;15(1):230. doi: 10.1186/s13023-020-01486-6. PMID: 32605583; PMCID: PMC7329487.

Test Your Knowledge

QUESTION 1

Conversion to which amino acid is inhibited in PKU?

A. Tryptophan
B. Phenylalanine
C. Tyrosine
D. Proline

QUESTION 2

Which of the following should be avoided in the diet of someone diagnosed with PKU?

A. Strawberries
B. Chickpeas
C. Butter
D. White bread

QUESTION 3

What is the target range for phenylalanine levels in the blood of children less than 12 years of age?

A. 0 µmol/L
B. 50-100 µmol/L
C. 120-360 µmol/L
D. 120-600 µmol/L

Answers

Question 1: C - PAH converts phenylalanine to tyrosine. In PKU, PAH is unable to convert phenylalanine to tyrosine causing a buildup of phenylalanine in the body.

Question 2: B - Chickpeas, also known as garbanzo beans should be avoided in PKU patients as beans are high in phenylalanine

Question 3: C - Blood phenylalanine levels in children less than 12 should be maintained between 120-360 µmol/L. In children older than 12, blood phenylalanine levels should be maintained between 120-600 µmol/L.

MSKD Block

A GUIDE FOR GOUT

Authors: Samantha Gaidula and Savanna Shaw

What is Gout?

Gout is a form of arthritis that is characterized by the deposition of monosodium urate monohydrate (MSU) crystals in tissues. Accumulation of uric acid, a byproduct of purine metabolism, can form these crystals which then can deposit and cause pain and inflammation. This can often be seen with excessive urate concentrations in the blood, typically exceeding 400 μmol/L.

Disease Management

Risk factors for gout include obesity, chronic renal disease, chronic alcohol use, tumor lysis syndrome and diets high in animal protein. To better manage gout, these items should be avoided or managed while attempting to achieve adequate protein consumption through plant sources. For acute flares, adequate hydration, NSAID usage, and systemic glucocorticoids are often used for pain and inflammation management.

A Note on Protein Sources

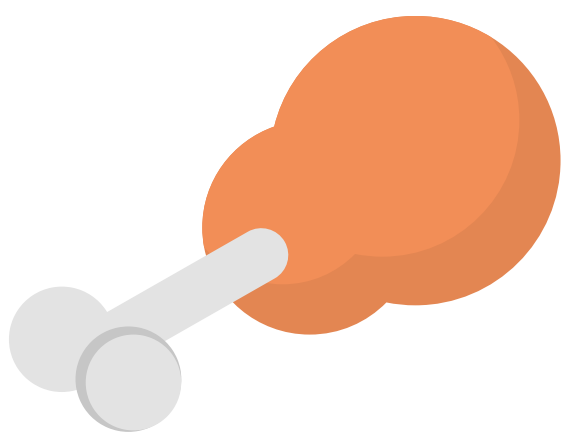

Uric acid production is increased by the consumption of foods high in purines such as organ meats and seafood. Plant based proteins and dairy protein sources should be substituted as they may have decreased uric acid production and can decrease the risk of gout.

Purine Metabolism

Purines → Hypoxanthine → Xanthine → Uric Acid

(Hypoxanthine → Xanthine: Xanthine Oxidase; Xanthine → Uric Acid: Xanthine Oxidase)

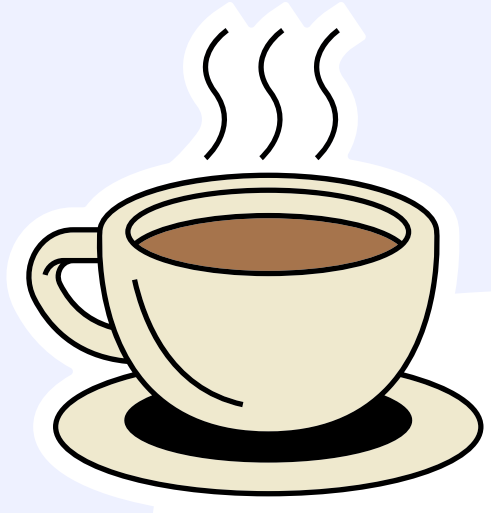

Grocery List Suggestions

Recommended:
- Fruits and vegetables
- Whole grains
- Skim milk (cow-based)
- coffee
- Varied protein sources

Limit:
- Game and red meats
- High fructose corn syrup
- Alcohol
- Certain seafoods (scallops, tuna, trout, etc.)

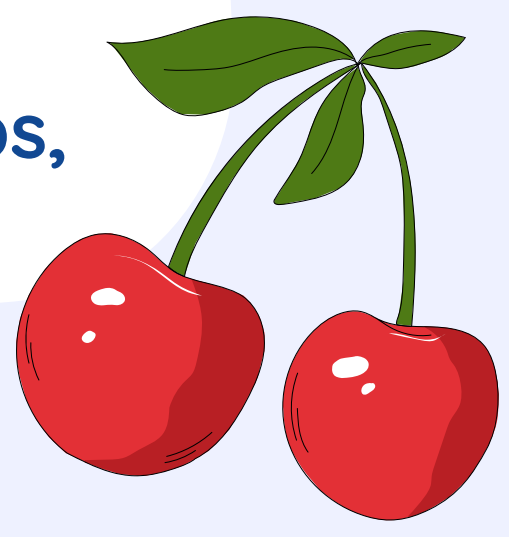

Recipe for Success

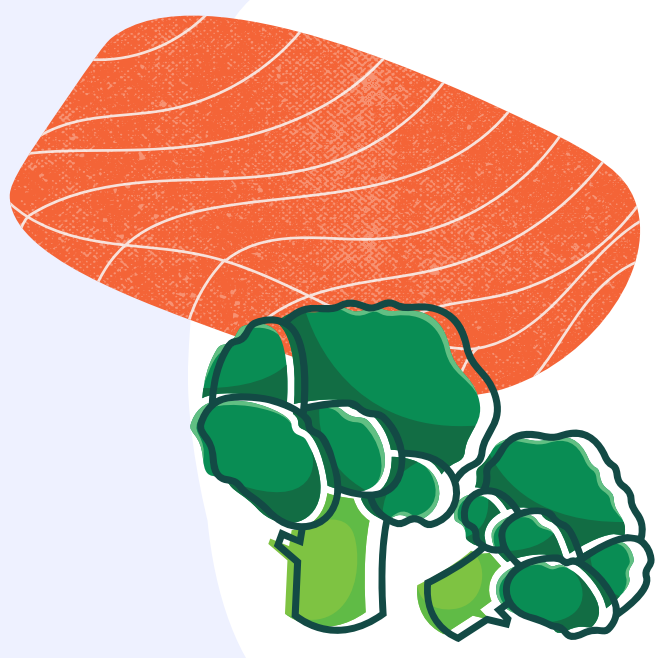

Breakfast: Low-fat Greek yogurt topped with blueberries, strawberries, and sliced almonds with a cup of coffee

Lunch: leafy green salad with tomatoes, bell peppers and grilled chicken breast with olive oil, lemon, salt, pepper and oregano

Dinner: baked salmon (moderate portion size), steamed broccoli and sweet potatoes

A Word on Skim Milk (Cow-based)

Numerous studies have shown an inverse relationship between serum uric acid levels and dairy product consumption; one particularly showed that skim milk consumption had a significant reduction in serum uric acid levels. One explanation for this association is that milk contains orotic acid which promotes renal urate excretion

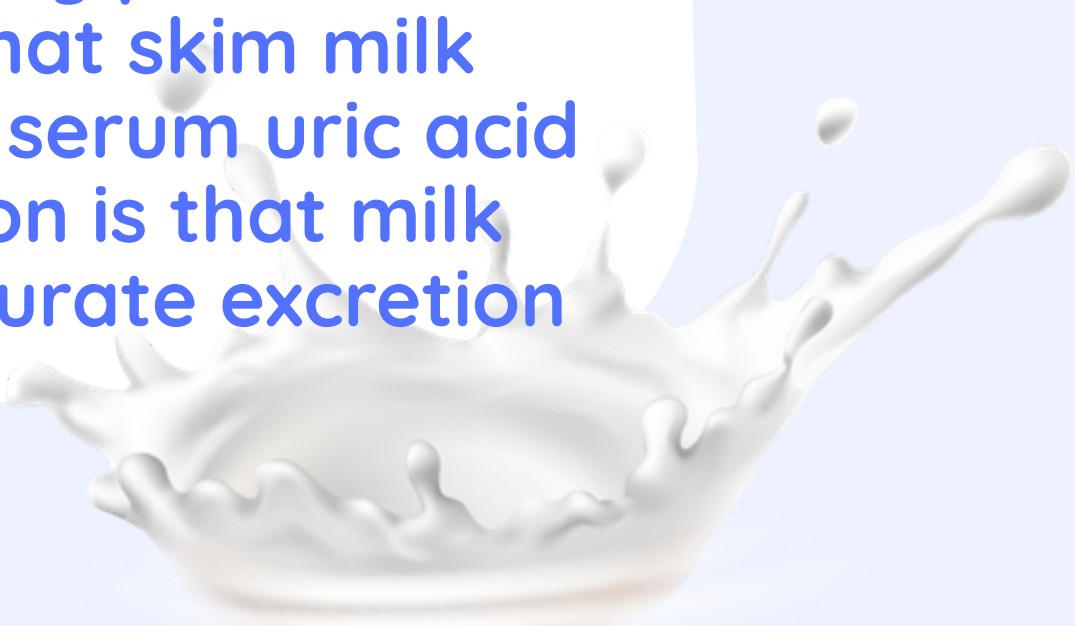

Yokose C, McCormick N, Choi HK. Dietary and Lifestyle-Centered Approach in Gout Care and Prevention. Curr Rheumatol Rep. 2021 Jul 1;23(7):51. doi: 10.1007/s11926-021-01020-y. PMID: 34196878; PMCID: PMC9281000.

Cleveland Clinic. "Gout Low Purine Diet." Cleveland Clinic, https://my.clevelandclinic.org/health/treatments/22548-gout-low-purine-diet

Towiwat P, Li ZG. The association of vitamin C, alcohol, coffee, tea, milk and yogurt with uric acid and gout. Int J Rheum Dis. 2015 Jun;18(5):495-501. doi: 10.1111/1756-185X.12622. PMID: 26082349.

Fenando A, Rednam M, Gujarathi R, Widrich J. Gout. 2024 Feb 12. In: StatPearls [Internet]. Treasure Island (FL): StatPearls Publishing; 2025 Jan–. PMID: 31536213.

Test Your Knowledge

QUESTION 1

Which of the following should NOT be a main part of the diet for someone experiencing gout?

A) Red meat
B) Cereal
C) Coffee
D) Asparagus

QUESTION 2

Which protein source has been shown to decrease the risk for gout?

A) Liver
B) 1% Milk
C) Chicken Breast
D) Pork Chop

QUESTION 3

Which metabolite would build up in the blood if the key enzyme in uric acid production were mutated?

A) Xanthine
B) Uric acid
C) Hypoxanthine

Answer Key

Question 1: A - Red meat is high in purines which breaks down into uric acid. Gout is a condition where uric acid levels are high so someone with gout would NOT want to consume products that could contribute to uric acid accumulation.

Question 2: B - Dairy sources of protein have been linked to a decrease in gout flare ups because they have less purines than meat sources of protein, such as those in the other answer choices.

Question 3: C - Xanthine oxidase is the enzyme used to convert both hypoxanthine to xanthine and xanthine to uric acid. If this enzyme does not work, the metabolites before it in the pathway would build up. This includes hypoxanthine and purines.

NUTRIMENT

GI Block

A GUIDE FOR CELIAC DISEASE

Authors: Samantha Gaidula and Savanna Shaw

What is Celiac Disease?

Celiac disease is an autoimmune condition in the small intestine that is triggered by the protein gluten which is found in wheat, rye, and barley. Celiac disease can manifest as a systemic disease, possibly causing skin rashes, malabsorption, abdominal discomfort, and osteoporosis. When gluten is ingested by a person with celiac disease, the small intestine produces an inflammatory reaction that results in villous atrophy, crypt hyperplasia, and immune cell infiltration of the lamina propria. This can lead to various vitamin deficiencies and health problems in the short term and long term for celiac disease patients[1]. Some individuals do not meet the criteria to be diagnosed with true celiac disease, but are considered to be celiac intolerant and manifest with many of these same signs and symptoms.

What makes someone susceptible?

Risk factors for celiac disease include genetics and environmental stimuli. Persons with the leukocyte histocompatibility antigens HLA-DQ2 and HLA-DQ8 serotypes are highly associated with celiac disease. According to Aboulaghras et al., 90% of celiac disease patients express the HLA-DQ2 haplotype, 5% carry the HLA-DQ8 haplotype, and the remaining 5% express a combination of both[2]. Other risk factors include being of European, Saudi Arabian or Saharawi dissent, biologically female, antibiotic use that disrupts the normal gut microbiome, pregnancy, and infections such as norovirus that may alter the body's inflammatory response[1].

Disease Management

Managing celiac disease starts by going completely gluten free to promote intestinal healing and symptom relief. Additionally, ensuring proper follow-up with a physician to avoid nutritional deficiencies and complications of celiac disease such as osteoporosis and skin manifestations is essential for physical, mental, and long-term health[1].

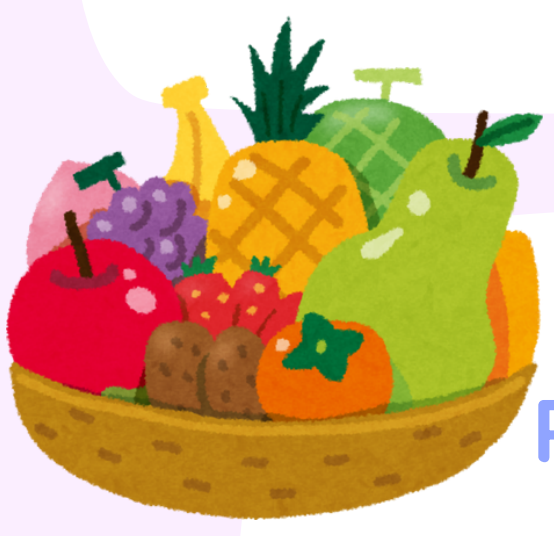

Grocery List Suggestions

Recommended:
- fruits
- vegetables
- unprocessed meats
- quinoa

Limit:
- wheat (pasta)
- barley (certain soups)
- rye (bread)
- oats
- beer

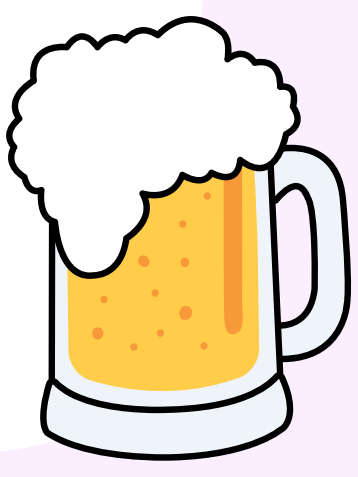

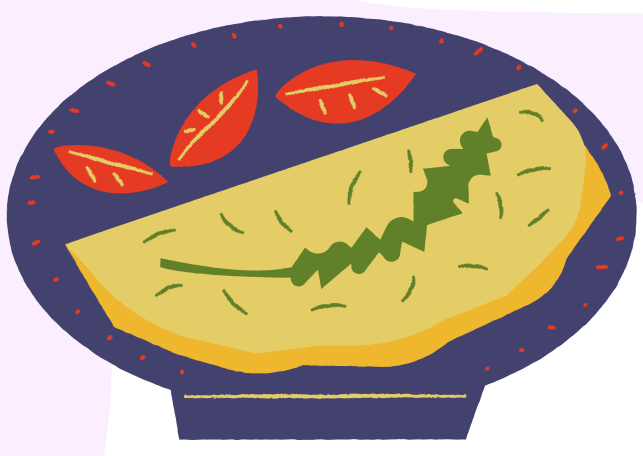

Recipe for Success

Breakfast: spinach and feta omelet with gluten free toast and a side of berries

Lunch: grilled chicken over quinoa with lettuce, gluten free croutons and caesar dressing

Dinner: Roasted salmon filet over brown rice with a side of carrots, zucchini and red bell peppers

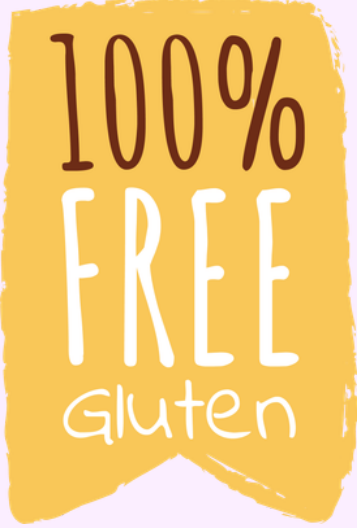

A Word on Cross Contamination

Cross contamination, or when a compound is transferred from one substance to another, is a major struggle for patients with celiac disease. Although many foods or restaurants may advertise themselves as gluten free, this may not be reality. For example, one study found that gluten was detected in 32% of gluten free labeled foods. To aid this major concern, gluten free detectors and testing kits are available and should be used when grocery shopping or out to eat.

- Daley SF, Haseeb M. Celiac Disease. 2025 Feb 4. In: StatPearls [Internet]. Treasure Island (FL): StatPearls Publishing; 2025 Jan–. PMID: 28722929.
- Aboulaghras S, Piancatelli D, Taghzouti K, Balahbib A, Alshahrani MM, Al Awadh AA, Goh KW, Ming LC, Bouyahya A, Oumhani K. Meta-Analysis and Systematic Review of HLA DQ2/DQ8 in Adults with Celiac Disease. Int J Mol Sci. 2023 Jan 7;24(2):1188. doi: 10.3390/ijms24021188. PMID: 36674702; PMCID: PMC9863503.
- Pinto-Sanchez MI, Blom JJ, Gibson PR, Armstrong D. Nutrition Assessment and Management in Celiac Disease. Gastroenterology. 2024 Jun;167(1):116-131.e1. doi: 10.1053/j.gastro.2024.02.049. Epub 2024 Apr 7. PMID: 38593924.
- Aljada B, Zohni A, El-Matary W. The Gluten-Free Diet for Celiac Disease and Beyond. Nutrients. 2021 Nov 9;13(11):3993. doi: 10.3390/nu13113993. PMID: 34836247; PMCID: PMC8625243
- Lerner BA, Phan Vo LT, Yates S, Rundle AG, Green PHR, Lebwohl B. Detection of Gluten in Gluten-Free Labeled Restaurant Food: Analysis of Crowd-Sourced Data. Am J Gastroenterol. 2019 May;114(5):792-797. doi: 10.14309/ajg.0000000000000202. PMID: 30920417; PMCID: PMC6502671

Test Your Knowledge

QUESTION 1

Which of the following is safe to eat for someone following a gluten free diet?

A) Beer
B) Pasta
C) Potatoes
D) Blueberry muffin

QUESTION 2

Which of the following is NOT a form of cross contamination?

A) Food to food
B) Equipment to food
C) Person to food
D) Person to equipment

QUESTION 3

Which haplotype is most associated with the onset of celiac disease?

A) HLA-DQ2
B) HLA-DQ4
C) HLA-DQ8

Answer Key

Question 1: C - Potatoes are considered a gluten free food and are safe to eat on a gluten free diet. Beer, pasta and muffins all potentially contain gluten.

Question 2: D- Person to equipment contamination such as a person who ate gluten who then touches a knife to prepare a meal is NOT considered a form of cross contamination. Cross contamination occurs when either a glutenous food touches a non-glutenous food, a piece of kitchen equipment that was used on a glutenous food is then used to prepare a non-glutenous food or when a person touches gluten and then handles a non-glutenous food.

Question 3: A -90% of all celiac disease patients express the HLA-DQ2 haplotype. While not everyone that has the HLA-DQ2 haplotype will develop celiac disease, those with the disease often have this gene.
